# Update of dialysis initiation timing in end stage kidney disease patients: is it a resolved question? A systematic literature review

**DOI:** 10.1186/s12882-023-03184-4

**Published:** 2023-06-07

**Authors:** Xiaoyan Jia, Xueqing Tang, Yunfeng Li, Dongmei Xu, Paulo Moreira

**Affiliations:** 1 Department of Nephrology, the First Affiliated Hospital of Shandong First Medical University & Shandong Provincial Qianfoshan Hospital, Jinan, 250014 China; 2 Shandong Institute of Nephrology, No.16766 Jingshi Road, Jinan, 250014 China; 3 Department of Nursing, the First Affiliated Hospital of Shandong First Medical University & Shandong Provincial Qianfoshan Hospital, Jinan, 250014 China; 4 International Healthcare Management Research & Development Centre, Shandong Provincial Qianfoshan Hospital AND Atlantica Instituto Universitario, Gestao em Saude, Oeiras, Portugal, Jinan, 250014 China

**Keywords:** Chronic kidney disease (CKD), End stage kidney disease (ESKD), Estimated glomerular filtration rate (eGFR), Optimal initiation of maintenance dialysis, Dialysis, Clinical practice guidelines, Systematic literature review

## Abstract

**Background:**

The exact optimal timing of dialysis for ESKD patients remains unknown. This study systematically reviewed the available evidence with regard to the optimal initiation of maintenance dialysis in ESKD patients.

**Methods:**

An electronic search was performed in Embase, PubMed and the Cochrane Library in order to find studies investigating associations between variables reference to “start of dialysis” and outcomes. Quality assessment and bias assessment were performed by the Newcastle–Ottawa scale and the ROBINSI tool. Due to the heterogeneity of studies, a meta-analysis could not be performed.

**Results:**

Thirteen studies were included; four studies included only haemodialysis patients, three peritoneal dialysis, six both; study outcomes included mortality, cardiovascular events, technique failure, quality of life and others. Nine studies mainly focused on the optimal GFR of maintenance dialysis initiation; five studies showed none association between GFR and mortality or other adverse outcomes, two studies showed dialysis initiation at higher GFR levels were with poor prognosis, and 2 studies showed higher GFR levels with better prognosis. Three studies paid attention to comprehensive assessment of uremic signs and/or symptoms for optimal dialysis initiation; uremic burden based on 7 uremic indicators (hemoglobin, serum albumin, blood urea nitrogen, serum creatinine, potassium, phosphorus, and bicarbonate) were not associated with mortality; another equation (combination of sex, age, serum creatinine, blood urea nitrogen, serum albumin, haemoglobin, serum phosphorus, diabetes mellitus, and heart failure) based on fuzzy mathematics to assess the timing of haemodialysis initiation was accuracy to prognose 3-year survival; the third study found that volume overload or hypertension was associated with the highest risk for subsequent mortality. Two studies compared urgent or optimal start in dialysis, a study reported increased survival in optimal start patients, another reported no differences between Urgent-Start-PD and Early-Start-PD regarding 6-month outcomes. Limitations: Heterogeneity among the studies was quite high, with differences in sample size, variable and group characteristics; no RCT studies were included, which weakened the strength of evidences.

**Conclusions:**

The criteria for dialysis initiation were varied. Most studies proved that GFR at dialysis initiation was not associated with mortality, timing of dialysis initiation should not be based on GFR, assessments of volume load and patient’s tolerance to volume overload are prospective approaches.

**Supplementary Information:**

The online version contains supplementary material available at 10.1186/s12882-023-03184-4.

## Introduction

Chronic kidney disease (CKD) is a major global public health problem [1], and there is an increasing number of end stage kidney disease (ESKD) patients start dialysis annually. The decision on optimal initiation of maintenance dialysis is a common problem faced by nephrologists. During 1980s-2000s, extensive observational studies have been attempted to investigate the optimal estimated glomerular filtration rate (eGFR) at the start of dialysis. In 2010 a randomized controlled trial named “trial Initiating Dialysis Early and Late (IDEAL)” showed that a strategy of early dialysis initiation (target eGFR: 10-14 mL/min/1.73m2) was not superior to late initiation (waiting until symptoms develop or eGFR is 5-7 mL/min/1.73m2) [2]. Since then, clinical practice guidelines suggest that the decision of initiate maintenance dialysis should be guided primarily by clinical constellation of signs and symptoms attributable to uremic syndrome [3–6]. However, symptoms or signs of uremia are varied and complex, mainly depends on clinical judgment; what’s more, typical uremic symptoms such as pericarditis and encephalopathy in patients without volume overload often occur at a very low GFR, these conditions are often combined with severe metabolic disorders and/or organ damages; the exact optimal timing of dialysis for ESKD patients remains unknown.

Therefore, the aim of our study is to systematically review the available evidence with regard to the optimal initiation of maintenance dialysis in ESKD patients.

## Methods

The systematic review adopted PRISMA protocol. The quality of studies was assessed through the Newcastle–Ottawa scale.

### Data source and search strategy

The PUBMED, EMBASE and COCHRANE databases were searched for English-language articles between Jan 2017 and Jan 2022, and with search terms as described in Supplementary data, Table S1.

### Study selection

Articles should meet any of the following criteria to be included: every study with reference to “start of dialysis” decision criteria that also (i) provided outcome data in patients; (ii) reported outcome data of an interaction analysis; (iii) there are predefined specific levels of kidney function at which RRT was initiated that were deemed early or late start of dialysis; (iv) RRT encompassed all forms of haemodialysis (HD) and peritoneal dialysis (PD); (v)editorials, case reports, reviews, letters and studies performed in children (age < 18 years) or animals should be excluded after screening for relevant references; (vi)primary study-objective was not the effect of the situation at the beginning of dialysis on prognosis, cross-sectional study, or study without follow-up results should be excluded.

### Quality and risk of bias assessment

Study quality was assessed by the Newcastle–Ottawa scale for observational studies, which provides a quality score per study based on three items: study participants (0–4 points), adjustment for confounding (0–2 points) and exposure/outcome of interest ascertainment (0–3 points). A study that meets all the criteria for these three dimensions would score a maximum of 9 points [7].Newcastle–Ottawa scale were assessed by two reviewers (Xiaoyan Jia and Xueqing tang), a third reviewer (Dongmei Xu) was consulted in case of doubt.

### Data extraction and analysis

After the selected titles were reviewed, two reviewers independently read the abstracts to select the relevant articles for full-text review. After this full-text review, the data in the full-text articles that met the inclusion criteria were extracted, tabulated and analyzed. Discrepancies between the two reviewers were solved by asking a third author to review the problematic articles and reach consensus. Two additional authors made a general revision of the whole text.

## Results

### Search results

**Fig. 1:**
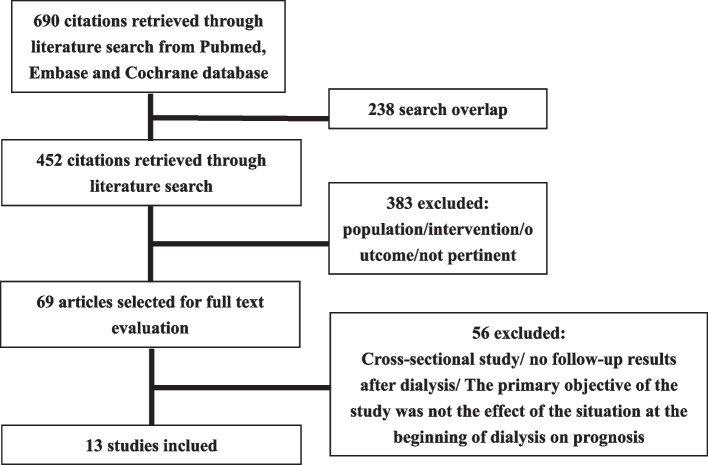
PRISMA flow chart for paper selection

Six hundred and ninety potentially relevant articles were retrieved by the search, and 238 articles were excluded due to search overlap. Based on title and abstract, 383 articles were excluded because the population or the exposure of the study did not meet our inclusion/exclusion criteria. A flow diagram of the article selection process is depicted in Fig. 1. Of the 69 studies selected for full-text examination, 56 were excluded because after full-text review it became apparent that the primary objective of these studies was not the effect of the situation at the beginning of dialysis on prognosis or due to cross-sectional study/no follow-up results after dialysis. Finally, 13 studies were reviewed in detail and included in this review. The characteristics of these studies can be found in Table 1.

**Table 1:** Characteristics and risk of bias of the included studies

Study	Publication year, time frame, location	Study design, n, (HD/PD), method	Inclusion and exclusion criteria	Intervention(n) and comparator(n), Outcome(s)	Baseline Patient characteristics	Results	Newcastle–Ottawa score and risk of bias	Notes
Martínez, A. C.et al. [8]	2019, 2006–2017, United states	retrospective cohort, *n* = 10,692, HD/PD, Kaplan–Meier method and Cox regression model	Inclusion: > 18 year-old patients who started renal replacement therapy; exclusion: Patients whose first option of treatment was the anticipated renal transplant; patients treated by domiciliary HD; patients who recovered renal function at some point of the monitoring; patients treated at some point outside Andalusia and who subsequently returned to the APHS; patients with indication of PD (ultrafiltration) for a refractory heart failure; or patients who had more than two changes of dialysis therapy	Optimal starts (*n* = 4937): definitive access to dialysis, planned dialysis start, a minimum of six-month follow-up by a nephrologist, and a first dialysis method coinciding with the one registered at 90 days Suboptimal starts (*n* = 5755) Outcome: survival rates	Mean age: 63.16 years, Gender: 61.63% male, mean Charlson Comorbidity Index 5.36 in Optimal group Mean age: 63.07 years, Gender: 63.3% male, mean Charlson Comorbidity Index 5.95 in Suboptimal group	Death of Optimal group was 35.62%, Death of Suboptimal group was 47.13%. Patients achieved optimal starts of renal replacement therapy showed higher survival rates (HR 0.669; 95% CI 0.628–0.712) in the multivariate analysis of Cox regression model	7	It would be desirable and necessary to increase the resources allocated to primary care of patients with CKD
N. Prasad et.al. [9]	2017, 2006–2010, Indian	observational prospective cohort, *n* = 352, PD, Kaplan–Meier analysis and Cox proportional hazards model	Patients initiated on CAPD as an initial modality of RRT were included, excluded patients with an age of < 18 years	Group 1 (mGFR ≤ 5, *n* = 92), Group 2 (mGFR > 5–10, n-146), Group 3 (mGFR > 10, = 114) Follow up until December 2014 Outcomes: all-cause mortality and technique failure	Age: 53.7 ± 11.7 years, Gender:72.8% male, mGFR 2.92 ± 1.24 ml/min/1.73 m2, Comorbidity score of Davies0.9 ± 0.7in group 1 Age: 51.1 ± 15.6 years, Gender:71.9% male, mGFR 7.79 ± 1.30 ml/min/1.73 m2, Comorbidity score of Davies 0.7 ± 0.7 in group 2 Age: 49.1 ± 11.85 years, Gender:72.8% male, mGFR 12.2 ± 1.68 ml/min/1.73 m2, Comorbidity score of Davies 0.8 ± 0.8 in group 3	Patient survival and technique survival were better in higher baseline GFR groups. Compared to Group 3, both Group 1 (HR—3.42, 95% CI—1.85–6.30, *P* = 0.000) and Group 2 (HR—2.16, 95% CI—1.26–3.71, *P* = 0.005) had higher risk of mortality. For technique survival, initial mGFR of ≤ 5 ml/min/1.73 m2 was significant risk factor for discontinuation of PD as compared to others (Group 1 vs. Group 3; HR—3.42, 95% CI—1.63–7.15, *P* = 0.001 and Group 1 vs. Group 2; HR—2.83, 95% CI—1.83–4.33, *P* = 0.004)	7	The patients with higher RRF at PD initiation have better nutritional status on follow-up
Fu, E. L.et al. [10]	2021, 2007–2016, Sweden	observational retrospective cohort, *n* = 10,290, HD/PD, A weighted pooled logistic regression model	Included criteria: Age 18 years or older; an eGFR measurement between 10 and 20 mL/min/1.73 m2, with a previous eGFR measurement between 10 and 30 mL/min/1.73 m2 as confirmation; no history of kidney replacement therapy; and at least one available measurement of systolic blood pressure, diastolic blood pressure, total calcium, phosphate, albumin, and haemoglobin	Compared 15 dialysis initiation strategies with eGFR values ranging between 4 and 19 mL/min/1.73m2 in increments of 1 mL/min/1.73 m2, and took an eGFR between 6 and 7 mL/min/1.73 m2 as the reference group Outcomes: Five-year all-cause mortality and major adverse cardiovascular events	Median age of 73 years, 35.7% were women, and 42.1% had diabetes. The median eGFR was 16.8 (14.3–18.6) mL/min/1.73m2	A parabolic relation was observed for mortality, with the lowest risk for eGFR15-16, Compared with dialysis initiation at eGFR6-7, initiation at eGFR15-16 was associated with a 5.1% (95% confidence interval 2.5% to 6.9%) lower absolute five-year mortality risk and 2.9% (0.2% to 5.5%) lower risk of a major adverse cardiovascular event, corresponding to hazard ratios of 0.89 (95% confidence interval 0.87 to 0.92) and 0.94 (0.91 to 0.98), respectively. This 5.1% absolute risk difference corresponded to a mean postponement of death of 1.6 months over five years of follow-up	8	Very early dialysis initiation may not outweigh the burden of a substantially longer period spent on dialysis for most patients
Liu, Y.et al. [11]	2020, 2009–2014, China	observational retrospective cohort, *n* = 1674, HD, Multivariate logistic regression analysis and Kaplan–Meier survival curves, log-rank tests, and multivariate Cox regression models	Inclusion criteria: 16–80 years old; CKD with eGFR < 30 mL/min/1.73 m2 within 3 months before dialysis; duration of maintenance hemodialysis > 3 months. Exclusion conditions: peritoneal dialysis or kidney transplantation before or after hemodialysis; the presence of cancer, chronic infection, liver cirrhosis, or other diseases that can affect survival time at the initiation of hemodialysis; died from nondisease-related causes; acute kidney disease; emergency hemodialysis	Patients were divided into 3 groups based on their eGFR at the initiation of dialysis (< 4 mL/min/1.73m2, *n* = 310; 4–8 mL/min/1.73m2, *n* = 1002; > 8 mL/min/1.73 m, *n* = 362) Outcomes: The survival time	Male patients accounted for 61.9%, patients with diabetes accounted for 31.9%, the average age was 53.4 ± 13.7 years old, the average eGFR at the start of dialysis was 6.5 ± 3.3 mL/min/1.73 m2	No significant difference was demonstrated among the 3 groups (< 4 mL/min/1.73 m2 was used as the reference, in comparison with 4–8 mL/min/1.73 m2 [*p* = 0.681] and > 8 mL/min/1.73 m2 [*p* = 0.403])	7	Being male, older, and having diabetes or heart failure before dialysis were the causes for early dialysis start
Silva, V. C. [12]	2020, 2016–2019, Brazil	retrospective cohort, *n* = 72, PD, Kaplan–Meier survival curves and log-rank tests	Inclusion criteria comprised adult patients that started PD therapy up to 14 days after catheter insertion	Urgent-start (US-PD) group (*n* = 40): Patients had an urgent indication of renal replacement therapy (RRT) and started PD within 72-h after catheter insertion Early-start (ES-PD) group (*N* = 32): PD initiated between 3 and 14 days Outcomes: First 30-day complications, 6-month hospitalization, and dropout rate	Age (years): Total 53.2 ± 15.2, US-PD 53.8 ± 16, ES-PD 52.8 ± 14.6; Male, n (%): Total 36 (50), US-PD 17 (53.1), ES-PD 19 (47.5); DM, n (%): Total 30 (42), US-PD 13 (40.6), ES-PD 17 (42.5)	No differences between USPD and ES-PD regarding demographic characteristics,30-day complications,6-month hospitalization, and dropout events were found. The most frequent short-term complication in patients who started PD urgently was leakage. The most common cause of dropout was transfer to HD	7	Considering the lack of available sites for HD around the country, PD is a safe treatment to overcome this deficiency
Nathaphop Chaichaya, et al. [13]	2020, 2011–2018, Thailand	Retrospective cohort study, *n* = 828, PD, Kaplan–Meier method and Cox regression model	Patients at least 15 years old with stage-5 CKD. Patients with incomplete data required for analysis were excluded	patients who had eGFR 3-month before starting PD > 5 ml/min/1.73m2 were included in the early-late group (*n* = 484), while those who had eGFR < 5 ml/min/1.73m2 were assigned in the very late group (*n* = 344) Outcomes: all-cause mortality and technique failure. Patient survival rates were calculated from the date of initiation of continuous PD until the date of death or up to 31 December 2018	Age: 56.1 ± 13.4 years, Gender: 52.3% male, eGFR 7.5 ± 2.5 mL/min/1.73 m2 at pre-dialysis in Early-late group Age: 55.9 ± 11.9 years, Gender: 42.4% male, eGFR 3.2 ± 0.8 mL/min/1.73 m2 at pre-dialysis in Very late group	Kaplan–Meier survival curves of all-cause mortality demonstrated a slightly lower median survival time (35 months) in early-late group than that in very late group (40 months). The median time to technique failure in both groups were similar (25 months). The log rank test of the two curves were not statistically significant different	7	Asymptomatic patients with stage-5 CKD may be safely managed by very late start PD plan and patients may benefit from the delayed PD initiation
Yun-Lun Chang, et al. [14]	2020, 2006–2015, China	Retrospective cohort study, *n* = 1079; HD; Cox regression analysis; mortality case-crossover; Each patient acted as his or her own control. The case period of each patient was defined as 0–30 days before dialysis initiation, and the matched control period was defined as 90–120 days before dialysis initiation; significant difference test was used to quantify uremic burden before dialysis initiation	Hemodialysis patients aged 18–90 years with continual care at China Medical University Hospital (CMUH) hemodialysis center between 2006 and 2015	1,079 patients, each patient acted as his or her own control. The case period of each patient was defined as 0–30 days before dialysis initiation, and the matched control period was defined as 90–120 days before dialysis initiation. Dialysis timing was classified as standard, late, and very late. Outcomes: mortality; followed up until December 31, 2016 or death, whichever occurred first	Age 61.4 (51.3, 71.2); 52% males; GFR 5.5 (4.1, 7.6) ml/min/1.73m2	Median eGFR-DI of the 1,079 patients was 3.4 mL/min/1.73 m2 and was 2.7 mL/min/1.73 m2 in patients with very late initiation. The median follow-up duration was 2.42 years. The fully adjusted hazards ratios of all-cause mortality for the late and very late groups were 0.97 (95% confidence interval 0.76–1.24) and 0.83 (0.61–1.15) compared with the standard group	7	It is safe to defer dialysis initiation among patients with an eGFR of < 5 mL/min/1.73 m2
Jae Yoon Park, et al. [15]	2017, 2008–2015, South Korea	Multicenter prospective cohort study; *n* = 665; HD/PD; Propensity score matching (PSM), Kaplan–Meier survival curves, Cox regression models	Patients aged 65 years or older who started dialysis for ESRD between July 2008 and February 2015 were eligible for the study	The patients were divided into 2 groups based on the median eGFR immediately prior to the initiation of dialysis: Early dialysis (*n* = 196), Late dialysis (*n* = 196) Outcomes: mortality; cardiovascular events and the 1-year changes in KDQOL-36survey, KPS values, BDI values, and SGA scores	Age (years): Early 71.7 ± 5.3, Late 72.1 ± 5.0; Male: Early 60.2%, Late 63.3%%; eGFR (ml/min/1.73m2): Early 12.5 ± 4.8, Late 7.0 ± 1.3	The survival rates of the 2 groups did not differ after adjusting for age, sex and so on. Although the early initiation group showed a lower physical component summary score on the KDQOL-36 3 months after dialysis, the difference in scores was not significant 12 months after dialysis. Furthermore, the difference was not significant after PSM. The Karnofsky performance scale, Beck’s depression inventory, and subjective global assessments were not significantly different 3 and 12 months after dialysis initiation	7 PSM was used to increase the precision of the estimated effect without increasing bias because certain variables were potentially associated with survival	This study did not consider the economic and ethical concerns regarding dialysis initiation; included only Korean patient; not RCT
Cynthia J Janmaat, et al. [16]	2017, 1997–2007, Netherlands	Multicenter prospective observational cohort study, *n* = 1143, HD/PD; Kaplan–Meier method, Cox proportional-hazard regression analyses;	Incident-dialysis patients aged ≥ 18 years with no history of renal replacement therapy (RRT; i.e., starting dialysis or renal transplantation) were included at the start of dialysis treatment. Patients were excluded when they had a hemodialysis catheter. The latter ensured we excluded patients with acute renal impairment	Tertiles of mGFR (Total *n* = 852) or eGFR (Total *n* = 1143) at moment of dialysis initiation (Late, Intermediate, Early) Outcomes: mortality; Patients were followed until time of death or censored, due to kidney transplantation, recovery of kidney function as reason to stop dialysis therapy, withdrawal from the study, transfer to a dialysis center that did not participate in the study, loss to follow-up, or end of the study period (February 2015), whichever came first	Late, intermediate, and early starters based on mGFR: Age (years) 62.1 (47.8–71.7), 61.8 (49.1–70.8), 59 (47.1–69.1); Male 60.2%, 62%, 63.4%; mGFR(ml/min/1.73m2) 2.5 ± 1.4, 5.4 ± 0.7, 8.9 ± 2.1 Late, intermediate, and early starters based on EGFR: Age (years) 58.3 (46.3–67.9), 62.5 (50.8–72), 66.3 (54.3–74.3); Male 52.8%, 63%, 72.2%; mGFR(ml/min/1.73m2) 4.4 (± 1.2), 6.7 (± 0.6), and 10.2 (± 2.3)	After lead-time correction, early initiation showed a survival disadvantage (HR between 1.1 [95% CI 0.82–1.48] and 1.33 [95% CI 1.05–1.68]). Dialysis start time differed about a year between early and late initiation	6 To examine the effect of lead-time bias, survival was counted from the time of dialysis initiation or from a common starting point (GFR 20 mL/min/1.73 m2), using linear interpolation models	Lead-time bias is not only a methodological problem but also has clinical impact when assessing the optimal kidney function to start dialysis
Rachele Escoli, et al. [17]	2017, 2010–2014, Portugal	Retrospective Cohort Study, *n* = 235,HD,Kaplan–Meier analysis,Cox regression analysis,Logistic regression	HD incident patients from 1 January 2010 to 30 September 2014	Patients were classified into two groups by estimated GFR at dialysis initiation (eGFR ≥ 10: early start, *n* = 42; < 10 mL/min per 1.73m2: late start, *n* = 193) Outcomes: mortality. Patient survival from the time of dialysis initiation to patient’s death or censor at 31 December 2015, which was the end of the study	Total: Age 70.7 ± 14.9 years;64.7% males; eGFR at dialysis initiation was 7.6 ± 3.8 ml/min per 1.73m2 Early start: Age 69.6 ± 17.1 years; 47.6% males; eGFR at dialysis initiation was 13.6 ± 4.8 ml/min per 1.73m2 Late start: Age 71 ± 14.4 years; 68.4% males; eGFR at dialysis initiation was 6.3 ± 1.8 ml/min per 1.73m2	Compared with the group with an eGFR of < 10 mL/min, independent factors (*P* < 0.05) associated with mortality in the multivariable Cox model in early dialysis start were: hypertension (HR 9.32, CI: 1.34–17.87), diabetes (HR 1.8, CI: 0.4–13.2) and albumin < 3.5 g/dL (HR 1.5, CI: 0.8–6.2). Older patients (HR 0.084, CI: 0.008–0.863) with low phosphorus levels (HR 0.02, CI: 0.0–0.527) also had statistically significant results, although they showed a reduced risk of mortality	6	Further research is needed to determine the objective signs, symptoms and laboratory test results associated with increased mortality and decreased quality of life among patients with advanced renal failure
Yumei Zhang, et al. [18]	2018, 2005–2014, China	Prospective observational cohort study; *n* = 294; HD; Kaplan–Meier analysis, Cox regression analysis	CKD undergoing treatment at the HD center of Shanghai Ninth People’s Hospital..Exclusion criteria included patients with acute kidney injury or patients with acute-on-chronic renal failure	Four groups based on eGFR(ml/min × (1.73 m2)-1): ≥ 10.5, *n* = 26; 8–10.4, *n* = 29; 6–8, *n* = 63; < 6, *n* = 176 Outcomes: all-cause mortality, cardiovascular events, such as cerebrovascular accidents along with ischemic stroke. The data were allied to mortality data from the medical records archive of the Ninth People’s Hospital through March 31, 2015	Mean (SD) age was 53.61(16.32); Male/female 189/105; The median eGFR of all patients at the start of hemodialysis was 5.43 (2.27–13.92) ml/min × (1.73 m2)-1	The multivariate Cox regression analysis indicated that CCI, cerebrovascular diseases and chronic obstructive pulmonary disease were significantly associated with all-cause mortality, but not eGFR at the dialysis initiation. Furthermore, stratified analyses confirmed elevated eGFR that had no advantage on long-term prognosis	7	Early initiation of dialysis did not provide a survival advantage for patients undergoing hemodialysis
Matthew B. Rivara, et al. [19]	2017, 2004–2012, Seattle	Retrospective Cohort Study, *n* = 461, HD/PD, Cox proportional hazards regression, all-cause mortality	All patients 18 years or older who initiated maintenance dialysis; patients were excluded for initiation of dialysis therapy at a non-UW-affiliated hospital or under the care of a non2UW-affiliated nephrologist and thus relevant data for dialysis therapy initiation decision making were not available	Patients were divided into four groups according to primary indication for dialysis: Lab Evidence of Kidney Function, *n* = 83; Uremic Symptoms, *n* = 216; Volume Overload/HTN, *n* = 95; Other/Unknown, *n* = 67 Outcomes: Follow-up for the primary outcome of all-cause mortality was available for study participants through December 31, 2013. Additional outcomes included transplantation, transfer to a nonaffiliated dialysis facility, and withdrawal from dialysis therapy, and event dates for these outcomes were obtained from dialysis facility electronic records	Mean (SD) age was 55(15); Male 63%; eGFR at dialysis initiation was 8.0 ± 4.2 ml/min per 1.73m2	Following adjustment for demographic variables, coexisting illnesses, and eGFR, initiation of dialysis therapy for uremic symptoms, volume overload or hypertension, or other/unknown reasons was associated with 1.12 (95% CI, 0.72–1.77), 1.69 (95% CI, 1.02–2.80), and 1.28 (95% CI, 0.73–2.26) times higher risk, respectively, for subsequent mortality compared to initiation for laboratory evidence of kidney function decline	6	Investigator assessment of symptoms present and determination of the primary indication for dialysis therapy initiation relied on clinical documentation, and while extensive, such documentation may be incomplete
Liang Feng, et al. [20]	2017, 2008–2011, Singapore	Retrospective Cohort Study, *n* = 3286, HD/PD, Cox proportional hazards regression, Kaplan–Meier approach, all-cause mortality	Analysis inclusion criteria consisted of Singaporean citizenship or permanent residency, initiation of dialysis between January 2008 and December 2011, and age ≥ 18 years at dialysis commencement. Patients recipients of a kidney transplant or with missing serum creatinine data were excluded	Early start: eGFR ≥ 10 ml/min/1.73 m2, *n* = 218; Intermediate start: eGFR 5 and 10 ml/min/1.73 m2, *n* = 1359; Late start: eGFR less than 5 ml/min/1.73 m2, *n* = 1709 Outcomes: mortality. The data was further linked with the National Death Registry to acquire survival information until December 2013	Mean (SD) age was 61.5 (12.7); Male/female 1862/1424; Median eGFR at dialysis initiation was 4.9 ml/min per 1.73m2	In the unadjusted analysis, both early and intermediate dialysis initiation groups were at greater risk of death relative to late dialysis (Early: HR = 2.47; Intermediate: HR = 1.54). In the multivariate model, a significant interaction was detected between age and eGFR at dialysis initiation (*p* = 0.04). Adjusted mortality risk progressively increased with earlier initiation of dialysis for patients aged 18–54 years (*p* = 0.006) and aged 55 to 64 years (*p* < 0.001),and no statistically significant difference was observed for patients aged 65 years or older (*p* = 0.12)	6	First, findings are subject to lead time bias, and indication bias. A second limitation is that serum creatinine measurement was not standardized. Third, adjusted HRs were computed using only 60% of the 3592 patients (*n* = 2148) due to missing data on covariates other than serum creatinine

### Characteristics of selected studies

Thirteen studies were reviewed, among which four were prospective [9, 15, 16, 18] and nine were retrospective cohort studies [8, 10–14, 17, 19, 20]. Six studies were published in 2017 [9, 15–17, 19, 20], one study was published in 2018 [18] and one in 2019 [8], four studies were published in 2020 [11–14] and the most recent in 2021 [10]. The smallest study included 72 patients [12] and the largest included 10 692 patients [8]. Four studies included only haemodialysis patients [11, 14, 17, 18], three studies only peritoneal dialysis patients [9, 12, 13] and the rest included both. All but two studies used Cox proportional hazards regression to calculate hazard ratios (HRs) for mortality. The study about urgent vs. early-start peritoneal dialysis by Silva et al. [12] was the one study that did not use Cox regression and used first 30-day complications, 6-month hospitalization events and 6-month dropout as outcomes. The nationwide cohort study from Sweden was the one study which estimated the effect of each dialysis initiation strategy on 5-year all-cause mortality and major adverse cardiovascular events by using a weighted pooled logistic regression model [10].

### Quality and risk of bias

For quality assessment, the Newcastle–Ottawa score of these 13 studies was 6 to 7. A detailed overview of the characteristics of the 13 studies is displayed in Table 1. In the absence of RCT studies, observational studies on timing of dialysis initiation face immortal time bias, selection/survivor bias, and lead time bias. Some studies provided strategies in the study design, data collection and statistical analysis phases to eliminate these biases; specifically, as follows: propensity score matching (PSM) [15], survival time counted from the time of dialysis initiation and/or from a common starting point (for example: GFR 20 mL/min/1.73m2) [16], target trial emulation using cloning, censoring and weighting [10]. By applying these methods, well conducted observational studies could provide strong evidences for clinical decisions. The possible causes of heterogeneity among study results were regional and ethnic discrepancy, and different types of dialysis, especially the disparate definitions of early dialysis. For example, some studies defined early dialysis in terms of GFR levels, while other studies are defined by a comprehensive determination of laboratory indicators (such as hemoglobin, serum albumin, blood urea nitrogen, serum creatinine, potassium, phosphorus, and so on) and/or clinical symptoms (including acute heart failure, pulmo-nary edema or hypertension that was difficult to control with medication).

### Study outcomes

#### Mortality

Four studies showed that patients with an optimal start of renal replacement therapy or patients who started dialysis at higher eGFR levels have a better survival. Martínez et al. [8] observed that these patients with an optimal start of dialysis (haemodialysis or peritoneal dialysis) have a greater survival than those who had a non-optimal start. The optimal start was defined when all the following criteria were met: a planned dialysis start, a minimum of 6-month follow-up by a nephrologist, and a first dialysis method coinciding with the one registered at 90 days. This result remained present and did not change materially after adjustment for sex, age, primary renal disease and dialysis modality [HRCrude = 0.635 (95% CI 0.598–0.674), *P* < 0.001 versus HRAdj = 0.669 (95% CI 0.628–0.712), < 0.001]. Matthew et al. [19] also found patients initiating dialysis therapy due to volume overload may have increased risk for mortality compared with patients initiating routine dialysis due to laboratory evidence of kidney function decline. Adjusting for demographic variables, coexisting illnesses, and estimated glomerular filtration rate, initiation of dialysis therapy for volume overload or hypertension was associated with 1.69 (95% CI, 1.02–2.80) times higher risk for subsequent mortality compared to initiation for laboratory evidence of kidney function decline.

Fu et al. [10] compared 15 dialysis initiation strategies with eGFR values ranging between 4 and 19 mL/min/1.73m^2^ in increments of 1 mL/min/1.73 m^2^ and observed that dialysis initiation at eGFR15-16 was associated with a 5.1% (95% CI 2.5% to 6.9%) lower absolute 5-year mortality risk compared with initiation at eGFR6-7. The 5.1% absolute risk difference corresponded to a mean postponement of death of 1.6 months over 5 years of follow-up. Prasad et al. [9] included exclusively patients with peritoneal dialysis in a prospective study and found that patient survival were better in higher baseline GFR groups. The 1-, 2-, 3-, and 5-year patient survival in patients with GFR ≤ 5 ml/min/1.73 m^2^ were 78.2%, 41.9%, 24.8%, and 7.8%; GFR between > 5 and 10 ml/min/1.73 m^2^ were 87.2%, 64.8%, 43.1%, and 19.1%; and GFR > 10 ml/min/1.73 m^2^ were 91.6%, 74.1%, 51.1%, and 20.2%; respectively. Compared to patients with GFR > 10 ml/min/1.73 m^2^, both patients with GFR ≤ 5 ml/min/1.73 m^2^ (HR—3.42, 95% CI—1.85–6.30, *P* = 0.000) and patients with GFR between > 5 and 10 ml/min/1.73 m^2^ (HR—2.16, 95% CI—1.26–3.71, *P* = 0.005) had higher risk of mortality in the adjusted model.

Escoli et al. [17] and Liang et al. [20] were the only studies observing higher all-cause mortality in the early starters. Escoli et al. [17] included just patients with haemodialysis and revealed early dialysis initiation with an eGFR of ≥ 10 mL/min per 1.73 m^2^ was associated with an increased mortality risk compared with the patients with an eGFR of < 10 mL/min per 1.73 m^2^ (*P* = 0.027), arguing against aggressive early dialysis initiation based primarily on eGFR alone. Independent factors (*P* < 0.05) associated with mortality in the multivariable Cox model in early dialysis start were: hypertension (HR 9.32, CI: 1.34–17.87), diabetes (HR 1.8, CI: 0.4–13.2) and albumin < 3.5 g/dL (HR 1.5, CI: 0.8–6.2). Liang et al. [20] found both early (eGFR ≥ 10 ml/min/1.73 m^2^) and intermediate (5 ml/min/1.73 m^2^ ≤ eGFR < 10 ml/min/1.73 m^2^) dialysis initiation groups were at greater risk of death relative to late (eGFR < 5 ml/min/1.73 m^2^) dialysis (Early: HR = 1.91; Intermediate: HR = 1.23) in adjusted analysis. The findings were further stratified by age and observed that early versus later initiation of dialysis was associated with significantly higher risk of mortality in Singapore’s non-elderly population, but not in patients aged 65 years or older (*p* = 0.12).

The remaining six studies all showed that the early start of dialysis had no association with the prognosis of survival, of which three from Chinese HD patients. Liu et al. [11] demonstrated that after adjusting for effectors of age, gender, diabetes, type of vascular access at initiation, clinical signs, and/or symptoms at the initiation of dialysis, and serum albumin, there was no significant difference in survival rate between the 3 groups (< 4 mL/min/1.73 m^2^ was used as the reference, in comparison with 4–8 mL/min/1.73 m^2^ [*p* = 0.681] and > 8 mL/min/1.73 m^2^ [*p* = 0.403]). Chang et al. [14] also found the fully adjusted hazards ratios of mortality for the late (3–5indicators) and very late (6–7 indicators) groups were 0.97 (95% confidence interval 0.76–1.24) and 0.83 (0.61–1.15) compared with the standard (met 0–2 uremic indicators) group. The 7 uremic indicators that reached the predefined threshold in case period, namely hemoglobin, serum albumin, blood urea nitrogen, serum creatinine, potassium, phosphorus, and bicarbonate. So the study concluded that it is safe to defer dialysis initiation among patients with CKD having an eGFR of < 5 mL/min/1.73 m^2^ even when patients having multiple biochemical uremic burdens. Zhang et al. [18] in a prospective observational cohort study indicated that Charlson comorbidity index, cerebrovascular diseases and chronic obstructive pulmonary disease were significantly associated with mortality, but not eGFR at the dialysis initiation by multivariate Cox regression analysis. Furthermore, stratified analyses confirmed elevated eGFR that had no advantage on long‑term prognosis. Therefore, the long‑term prognosis of patients with high eGFRs prior to hemodialysis was not improved.

For PD patients, Nathaphop et al. [13] found that a slightly lower median survival time (35 months) in early-late (eGFR > 5 ml/min/1.73m^2^) group than that in very late (eGFR < 5 ml/min/1.73m^2^) group (40 months), but the difference of the two groups were not statistically significant (*p* = 0.56). Park et al. [15] conducted a multicenter prospective cohort study in HD/PD elderly patients and observed that the cumulative survival rates were lower in the early initiation group, but the difference was not significant after propensity score matching (PSM) or adjusting for age, sex, Charlson comorbidity index and hemoglobin, serum albumin, serum calcium and phosphorus levels. Janmaat et al. [16] also found taking account of lead-time bias, early dialysis initiation (eGFR > 7.9, measured GFR (mGFR) > 6.6 mL/min/1.73 m^2^) was not associated with an improvement in survival in HD/PD patients and suggested that in some patients, dialysis could be started even later than an eGFR < 5.7 and mGFR < 4.3 mL/min/1.73 m^2^.

#### Cardiovascular events

Cardiovascular events included cardiovascular death, non-fatal myocardial infarction, admission for ischemic heart disease, congestive heart failure, arrhythmia, or cerebrovascular disease. Fu et al. [10] showed that compared with dialysis initiation at eGFR6-7, initiation at eGFR15-16 was associated with a 2.9% (0.2% to 5.5%) lower risk of a major adverse cardiovascular event, corresponding to hazard ratios of 0.94 (0.91 to 0.98) and concluded that very early initiation of dialysis was associated with a modest reduction in cardiovascular events. However, Park et al. [15] indicated that differences in the cumulative cardiovascular event-free survival rates between early and late dialysis groups in elderly Korean patients were not observed before and after PSM.

#### Technique failure

Prasad et al. [9] and Nathaphop et al. [13] investigated technique failure on peritoneal dialysis. Prasad et al. [9] found initial mGFR of ≤ 5 ml/min/1.73 m^2^ was significant risk factor for discontinuation of PD as compared to others (vs. GFR > 10 ml/min/1.73 m^2^; HR—3.42, 95% CI—1.63–7.15, *P* = 0.001 and vs. GFR between > 5 and 10 ml/min/1.73 m^2^; HR—2.83, 95% CI—1.83–4.33, *P* = 0.004). Therefore, initiation of PD at a lower baseline mGFR is associated with poorer technique survival in Indian ESKD patients. On the contrary, Nathaphop et al. [13] observed that the median time to technique failure in early-late group and very late group were similar (25 months). The log rank test of the two curves were not statistically significant different.

#### Quality of life and other outcomes

Park et al. [15] also investigated the 1-year changes in the Kidney Disease Quality of Life-36 (KDQOL-36) survey, KPS values, Beck’s depression inventory (BDI) values, and SGA scores in elderly dialysis population. They showed that although the early initiation group showed a lower physical component summary score on the KDQOL-36 3 months after dialysis, the difference in scores was not significant 12 months after dialysis. Furthermore, the difference was not significant after PSM. The Karnofsky performance scale, Beck’s depression inventory, and subjective global assessments were not significantly different 3 and 12 months after dialysis initiation.

Silva et al. [12] just compared 30-day complications and 6-month hospitalization and dropout rate in patients that started PD therapy defined as urgent and early-start and found no differences between Urgent-Start-PD and Early-Start-PD regarding first 30-day complications, 6-month hospitalization, and dropout events were found.

## Discussion

Currently, clinical practice guidelines [3–6] recommend that: decision to initiate maintenance dialysis primarily depends on clinical signs and symptoms which may be attributed to uremic syndrome. In this regard, the Improving Global Outcomes (KDIGO) CKD Work Group suggested that the decision is usually occurring within the GFR range between 5 and 10 ml/min/1.73 m2″ [3, 6]. The Additionally, the Canadian Society of Nephrology suggested that in the absence of these factors,the eGFR should only serve as a sole criterion for the initiation of dialysis if it is 6 mL/min/1.73m2 or less”; [4]. The Japanese Society for Dialysis Therapy proposes that patients endure under conservative treatment until the GFR < 8 mL/min/1.73m2, even if symptoms of renal failure are observed and hemodialysis is recommended to be initiated prior to a GFR of 2 mL/min/1.73 m2, [21]. Our present systematic review included 13 studies of the last 5 years which investigated optimal dialysis initiation in ESKD patients, 9 of the 13 studies mainly focused on the optimal GFR of maintenance dialysis initiation; 7 studies used eGFR [10, 11, 13, 15, 17, 18, 20], 1 study used mGFR [9], 1 study used both [16]. Compared to previous studies, wider range of GFR values and more detailed data stratification were applied in the study design and data processing stages; 7 studies [9–11, 13, 16, 20] taken GFR ≤ 5 mL/min/1.73 m2 or even lower into account, the lowest data range was mGFR < 4.3 mL/min/1.73m2 [16] and the highest range was eGFR 15–16 mL/min/1.73 m2 [10]. 5 studies [11, 13, 15, 16, 18] showed none association between GFR and mortality or other adverse outcomes, 2 studies [17, 20] showed dialysis initiation at higher GFR levels were with poor prognosis, and 2 studies [9, 10] showed higher GFR levels with better prognosis. However, it’s worth noting that Fu et al. [10] observed a parabolic relation between eGFR and mortality, with the lowest mortality risk at eGFR15-16 mL/min/1.73m2; compared with dialysis initiation at eGFR6-7 mL/min/1.73m2, initiation at eGFR15-16 mL/min/1.73m2 was associated with a 5.1% lower absolute 5 year mortality risk and 2.9% lower risk of a major adverse cardiovascular; this 5.1% absolute risk difference corresponded to a mean postponement of death of 1.6 months over 5 years of follow-up, and dialysis would need to be started 4 years earlier; so they concluded that “although very early dialysis initiation was associated with a modest reduction in mortality and cardiovascular events, this may not outweigh the burden of a substantially longer period spent on dialysis. In recent years there has been an increase in the prevalence of elderly population, which, along with advances in dialysis technology and increasing survival of maintenance dialysis patients, lead to an increasing proportion of elder dialysis patients [22–24]. Two studies researched timing of dialysis initiation in elderly ESKD patients, and they both concluded that eGFR at dialysis initiation was not associated with mortality [15, 20] and quality of life [15]. So to summarize, most studies proved that GFR at dialysis initiation was not associated with mortality, timing of dialysis initiation should not be based on GFR both in all maintenance dialysis population and in elderly, which is consistent with present clinical guidelines.

In patients with ESKD, because of artificial low plasma creatinine levels in patients with fluid overload or low muscle mass, eGFR is falsely overestimated compared to their true GFR, some guidelines recommend mGFR instead of creatinine-based eGFR equations as the measure of dialysis in ESKD patients [21, 25]. However, perhaps because mGFR is simpler and easier than eGFR in clinical practice, there were 2 studies involved mGFR and showed different results; Prasad et al. [9] concluded that “initiation of chronic ambulatory peritoneal dialysis at a lower baseline mGFR is associated with poorer patient and technique survival” in Indian ESKD patients; Janmaat et al. [16] summarized that early dialysis initiation (mGFR > 6.6 mL/min/1.73m2) was not associated with an improvement in survival in dialysis PD/HD patients. This finding is similar to statistic analysis based on eGFR. Based on only the above 2 studies, we can’t draw any conclusions on to what extent mGFR is essential to determine dialysis initiation in ESKD patients; however, this does not mean that the importance and reliability of mGFR in evaluating residual renal function of ESKD patients can be denied.

Hence, a number of other studies paid attention to comprehensive assessment of uremic signs and/or symptoms for optimal dialysis initiation. Chang et al. [14] quantified uremic burden based on 7 uremic indicators that reached the predefined threshold (hemoglobin, serum albumin, blood urea nitrogen, serum creatinine, potassium, phosphorus, and bicarbonate); dialysis timing was classified as standard (met 0–2 uremic indicators), late (3–5indicators), and very late (6–7 indicators); and no correlation was found between late or very late group and mortality. Ying et al. [26] developed an equation based on fuzzy mathematics to assess the timing of haemodialysis initiation, the results showed that the combination of sex, age, serum creatinine, blood urea nitrogen, serum albumin, haemoglobin, serum phosphorus, diabetes mellitus, and heart failure as equation variables resulted in the best accuracy to prognose 3-year survival. Matthew et al. [19] categorized clinically documented primary indication for dialysis initiation into 4 groups: laboratory evidence of kidney function decline (reference category), uremic symptoms, volume overload or hypertension, and other/unknown; and found that volume overload or hypertension was associated with the highest risk for subsequent mortality. These studies highlight future directions of comprehensive evaluation of dialysis timing, especially in patients with volume overload and complications sensitive to volume changes during dialysis, mainly varied causes of cardiovascular instabilities. It is well known that the main functions of PD or HD are to remove excess volumetric loads, filter solutes and balance electrolytes. As discussed above, previous studies about dialysis timing focused on GFR, which represent functions of solutes filter and electrolytes balance, showed no positive findings. Present novel finding is that: heart failure, volume overload or hypertension at dialysis initiation was associated with the highest risk for subsequent mortality; and suggests assessments of volume load and patient’s tolerance to volume overload as prospective approaches, especially in ESKD patients with cardiovascular disease. Meanwhile, gathering more symptoms and biological disturbances compared to a start only based on GFR in future study is probably of upmost importance.

Strengths of our study include: firstly, we performed a systematic search according to a strict methodology; secondly, we provided approaches and methods for investigation of optimal dialysis initiation. Limitations: firstly, heterogeneity among the studies was quite high, with differences in sample size, variable and group characteristics; secondly, no RCT studies were included, which weakened the strength of evidences; thirdly, the review was not registered and the protocol was not prepared.

Further studies dedicated to generate evidence on this topic, while assuming recent general proposals for healthcare research [27–29], should diversify and innovate namely through technological solutions, apps and platforms, becoming widely available to support trial designs. Electronic informed consent (eConsent) and web-based questionnaires are two new trial elements to influence methodologies. Adoption of decentralized and hybrid clinical research designs, electronic patient-reported outcome (ePRO) to generate real-world evidence (RWE) and real-world data (RWD) and the adoption of artificial intelligence (AI), Big Data, application programming interfaces (APIs) and digital platforms expected to improve patient selection, enhance data collection, integration and analysis, while at the same time reduce time, this level of digital transformation would improve both pre-clinical and clinical research, namely on improving the available evidence on optimal initiation of maintenance dialysis in end stage kidney disease patients.

## Conclusions

In conclusion, most studies proved that GFR at dialysis initiation was not associated with mortality, timing of dialysis initiation should not be based on GFR, assessments of volume load and patient’s tolerance to volume overload are prospective approaches.

## Supplementary Information

**Additional file 1: Table S1.** Search terms used in the PUBMED, EMBASE and COCHRANE databases.

## Data Availability

All data generated or analysed during this study are included in this published article [and its supplementary information files].
