# Supplementary material for: Update of dialysis initiation timing in end stage kidney disease patients: is it a resolved question? A systematic literature review
Source: BMC Nephrol. 2023 Jun 7;24:162. doi: 10.1186/s12882-023-03184-4 (PMC10249158; doi:10.1186/s12882-023-03184-4)
Supplement: Supplementary file 1 — Additional file 1: Table S1. Search terms used in the PUBMED, EMBASE and COCHRANE databases. [file 12882_2023_3184_MOESM1_ESM.docx]

**Table S1. Search terms used in the PUBMED, EMBASE and COCHRANE databases**

| databases | Search terms | n. |
| --- | --- | --- |
| PUBMED | ((((((((((chronic kidney disease[Title/Abstract]) OR (uremia[Title/Abstract])) OR (end stage renal failure[Title/Abstract])) OR (chronic renal failure[Title/Abstract])) OR (chronic kidney disease 5[Title/Abstract])) OR (end stage kidney disease[Title/Abstract])) OR (end stage renal disease[Title/Abstract])) AND (((((outcomes[Title/Abstract]) OR (prognosis[Title/Abstract])) OR (outcome[Title/Abstract])) OR (survival[Title/Abstract])) OR (Mortality[Title/Abstract]))) AND (((((start[Title/Abstract]) OR (initiate[Title/Abstract])) OR (timing[Title/Abstract])) OR (begin[Title/Abstract])) OR (Initiation[Title/Abstract]))) AND (((((dialysis[Title/Abstract]) OR (hemodialysis[Title/Abstract])) OR (peritoneal dialysis[Title/Abstract])) OR (kidneys artificial[Title/Abstract])) OR (renal replacement therapy[Title/Abstract]))) NOT (((((((acute kidney injury[Title/Abstract]) OR (acute kidney disease[Title/Abstract])) OR (acute kidney failure[Title/Abstract])) OR (acute renal insufficiency[Title/Abstract])) OR (acute renal injury[Title/Abstract])) OR (acute renal failure[Title/Abstract])) OR (AKI[Title/Abstract])) Filters: Clinical Trial, Multicenter Study, Observational Study, Pragmatic Clinical Trial, Randomized Controlled Trial, in the last 5 years, Humans, English | 151 |
| EMBASE | ('chronic kidney disease':ti,ab,kw OR uremia:ti,ab,kw OR 'end stage renal failure':ti,ab,kw OR 'chronic renal failure':ti,ab,kw OR 'chronic kidney disease 5':ti,ab,kw OR 'end stage kidney disease':ti,ab,kw OR 'end stage renal disease':ti,ab,kw) AND (dialysis:ti,ab,kw OR hemodialysis:ti,ab,kw OR 'peritoneal dialysis':ti,ab,kw OR 'kidneys artificial':ti,ab,kw OR 'renal replacement therapy':ti,ab,kw) AND (start:kw OR initiate:kw OR timing:kw OR begin:kw OR initiation:kw OR start:ti OR initiate:ti OR timing:ti OR begin:ti OR initiation:ti) AND (outcomes:ti,ab,kw OR prognosis:ti,ab,kw OR outcome:ti,ab,kw OR survival:ti,ab,kw OR mortality:ti,ab,kw) AND (2017:py OR 2018:py OR 2019:py OR 2020:py OR 2021:py OR 2022:py) AND ('clinical article'/de OR 'clinical study'/de OR 'clinical trial'/de OR 'clinical trial topic'/de OR 'cohort analysis'/de OR 'comparative effectiveness'/de OR 'comparative study'/de OR 'control group'/de OR 'controlled study'/de OR 'cross sectional study'/de OR 'crossover procedure'/de OR 'diagnostic test accuracy study'/de OR 'double blind procedure'/de OR 'feasibility study'/de OR 'human'/de OR 'intermethod comparison'/de OR 'longitudinal study'/de OR 'major clinical study'/de OR 'medical record review'/de OR 'methodology'/de OR 'model'/de OR 'multicenter study'/de OR 'observational study'/de OR 'open study'/de OR 'outcomes research'/de OR 'pilot study'/de OR 'proportional hazards model'/de OR 'prospective study'/de OR 'qualitative research'/de OR 'randomized controlled trial'/de OR 'randomized controlled trial topic'/de OR 'retrospective study'/de OR 'statistical model'/de OR 'total quality management'/de OR 'trend study'/de) AND ('article'/it OR 'article in press'/it OR 'chapter'/it) NOT ('acute kidney injury':ti OR 'acute kidney failure':ti OR 'acute renal insufficiency':ti OR 'acute renal injury':ti OR 'acute renal failure':ti OR aki:ti OR 'acute kidney disease':ti) | 142 |
| COCHRANE | (chronic kidney disease) OR (uremia) OR (end stage renal failure) OR (chronic renal failure) OR (chronic kidney disease 5) OR (end stage kidney disease) OR (end stage renal disease) in Title Abstract Keyword AND (outcomes) OR (prognosis) OR (outcome) OR (survival) OR (Mortality) in Title Abstract Keyword AND (start) OR (initiate) OR (timing) OR (begin) OR (Initiation) in Title Abstract Keyword AND (dialysis) OR (hemodialysis) OR (peritoneal dialysis) OR (kidneys artificial) OR (renal replacement therapy) in Title Abstract Keyword NOT (acute kidney injury) OR (acute kidney disease) OR (acute kidney failure) OR (acute renal insufficiency) OR (acute renal injury) OR (acute renal failure) OR (AKI) in Title Abstract Keyword - with Cochrane Library publication date Between Jan 2017 and Jan 2022, in Trials | 397 |
